# 1-Hy­droxy-3,4-dimeth­oxy-10-methyl­acridin-9-one

**DOI:** 10.1107/S2414314620010056

**Published:** 2020-08-11

**Authors:** Rodrigo S. A. de Araújo, Ernestine N. T. Zondegoumba, Whisthler L. D. Tankoua, Barthelemy Nyassé, Francisco J. B. Mendonça-Junior, Carlos A. De Simone

**Affiliations:** a Laboratory of Synthesis and Drug Delivery, Center of Applied Biological and Social Sciences, State University of Paraiba-Campus V; 58071-160, João Pessoa, Paraiba, Brazil; b Laboratory of Medicinal Chemistry, Departament of Organic Chemistry, Faculty of Sciences, University of Yaounde I; POBOX 812, Yaounde, Cameroon; c Departamento de Física e Informática, Instituto de Física de São Carlos, Universidade de São Paulo - USP, 13560-970 - São Carlos, SP, Brazil; Universidade de Sâo Paulo, Brazil

**Keywords:** crystal structure, Zanthoxylum leprieurii, acridone derivative

## Abstract

There are two independent mol­ecules in the asymmetric unit of the title compound, C_16_H_15_NO_4_, which was isolated from fruits of Zanthoxylum leprieurii. The atoms of the three rings of each mol­ecule are close to coplanar with the largest deviations from the least-squares planes being 0.084 (3) Å and 0.069 (2) Å. Each molecule features an intramolecular O—H⋯·O hydrogen bond. In the crystal, C—H⋯·O hydrogen-bonding inter­actions link the mol­ecules into a three-dimensional network.

## Abstract 2

The title compound was isolated from fruits of Zanthoxylum leprieurii. The atoms of the three rings of the mol­ecule are coplanar. In the crystal, C—H⋯·O hydrogen-bonding inter­actions link the mol­ecules into a three-dimensional network.

## Graphical abstract

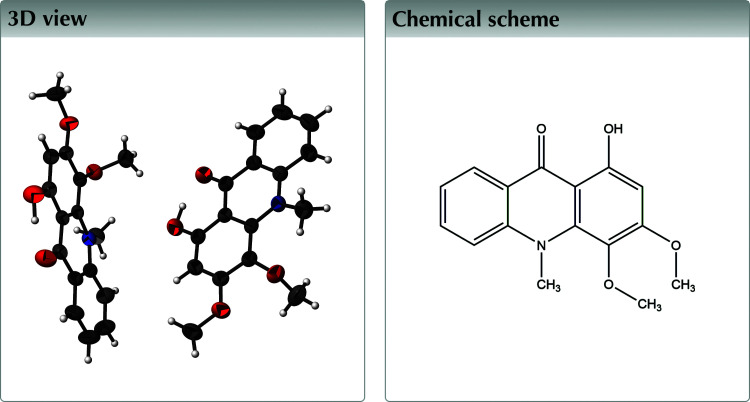

## Structure description

Zanthoxyllum leprieurii is one of the 549 species of the Zanthoxyllum genus (Rutaceae). It is widely distributed in tropical Africa (Wansi *et al.*, 2016 ▸), and is used in traditional medicine for the treatment of anaemia, arthritis, rheumatism, pain, leprosy, HIV, malaria, stomach problems and urinary diseases, as well as having vermifuge, diuretic and laxative properties (Guetchueng *et al.*, 2017 ▸). Its anti­cancer, anti­microbial, anti­plasmodial and anti­oxidant activities have also recently been well studied (Misra *et al.*, 2013 ▸). For its biological activity, see: Lamorde *et al.*, (2010); Ngane *et al.*, (2000). For related structures, see: Baudouin *et al.* (1985 ▸); Tchinda *et al.*, (2009).

One of the most important secondary metabolites described in Z. leprieurii are acridone derivatives (Ngoumfo *et al.*, 2010 ▸), of which we can highlight 1-hy­droxy-3,4-dimeth­oxy-*N*-methyl­acridone, which was first described by Baudouin *et al.* (1985 ▸) and has been isolated and evaluated for its bioactive potential in a single or synergistic action with other natural constituents (Baudouin *et al.*, 1985 ▸). For background to 1-hy­droxy-3,4-dimeth­oxy-*N*-methyl-acridone, see: Ladino & Suárez (2010 ▸). In this work, we describe the crystal structure of the title compound, isolated from Z. leprieurii.

**Figure 1:**
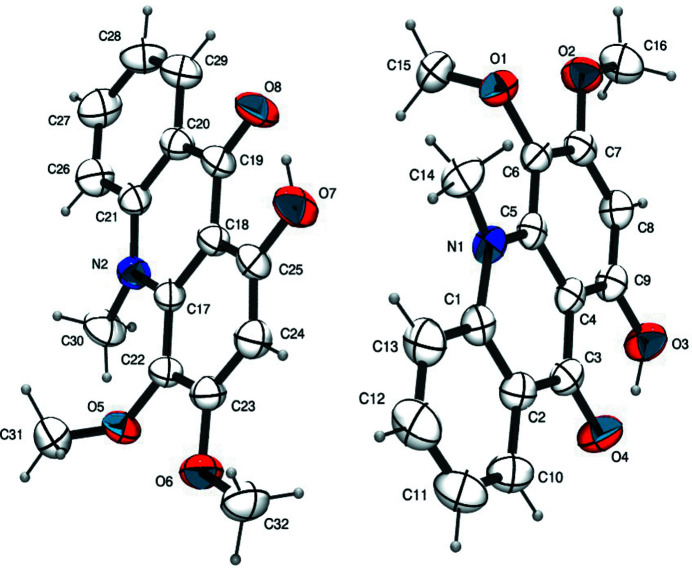
The asymmetric unit of the title compound showing the atom labelling and 50% probability displacement ellipsoids.

There are two independent mol­ecules in the asymmetric unit of the title compound, as shown in Fig. 1 ▸. The atoms of the three rings of the mol­ecule are close to coplanar, the largest deviations from their least-square planes being exhibited by atoms C12 [0.084 (3) Å] and C26 [0.069 (2) Å]. Atoms O1, O2, O3 and C16 lie close to the mean least-squares plane of the ring system with deviations of 0.068 (2), 0.053 (2), 0.067 (2) and 0.102 (2) Å, respectively [O5 −0.082 (1), O6 0.013 (2), O7 −0.120 (2), C32 0.033 (3) Å in the second independent mol­ecule]. Atoms C14 and C15 are −0.502 (2) and 1.374 (2) Å, respectively, out of the ring-system plane. The deviations for the second mol­ecule are 0.440 (2) for C30 and −1.409 (2) Å for C31. The outer rings make dihedral angles of 3.26 (8) and 2.46 (7) ° with the central ring in the first mol­ecule [2.84 (5) and 1.53 (4)° in the second.

In the crystal mol­ecules are linked by weak C—H⋯O inter­actions (Table 1 ▸).

## Synthesis and crystallization

The powder obtained after the pulverization of the plant material was soaked in a mixture of methyl­ene chloride and methanol. The crude extract was subjected to chromatography on a silica gel column eluted with an increasing polarity of ethyl acetate in hexane. The title compound, red in colour, soluble in acetone was obtained from fractions 111–120 (90 mg) by recrystallization from hexa­ne/ethyl acetate 80/20 solution. The solvent used for single-crystal formation was absolute ethanol.

Spectroscopic data the title compound are in agreement with literature data (Baudouin *et al.*, 1985 ▸).

## Refinement

Crystal data, data collection and structure refinement details are summarized in Table 2 ▸.

## Supplementary Material

Crystal structure: contains datablock(s) I, default. DOI: 10.1107/S2414314620010056/ex4002sup1.cif

Supporting information file. DOI: 10.1107/S2414314620010056/ex4002Isup2.cml

CCDC reference: 2018018

Additional supporting information:  crystallographic information; 3D view; checkCIF report

## Figures and Tables

**Table 1:** Hydrogen-bond geometry (Å, °)

*D*—H⋯*A*	*D*—H	H⋯*A*	*D*⋯*A*	*D*—H⋯*A*
C8—H8⋯O3^i^	0.93	2.52	3.441 (2)	170
C32—H32*A*⋯O8^ii^	0.96	2.55	3.460 (3)	157
C30—H30*C*⋯O7^iii^	0.96	2.59	3.513 (3)	159

Symmetry codes: (i) 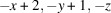 ; (ii) 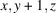 ; (iii) 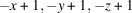 .

**Table 2:** Experimental details

Crystal data	
Chemical formula	C_16_H_15_NO_4_
*M*_r_	285.29
Crystal system, space group	Triclinic, *P* 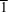
Temperature (K)	293
*a*, *b*, *c* (Å)	8.3360 (2), 10.1780 (3), 16.3260 (4)
α, β, γ (°)	82.284 (2), 76.557 (2), 87.081 (2)
*V* (Å^3^)	1334.73 (6)
*Z*	4
Radiation type	Mo *K*α
μ (mm^−1^)	0.10
Crystal size (mm)	0.36 × 0.23 × 0.15

Data collection	
Diffractometer	Nonius KappaCCD
No. of measured, independent and observed [*I* > 2σ(*I*)] reflections	19726, 6073, 4671
*R*_int_	0.022
(sin θ/λ)_max_ (Å^−1^)	0.649

Refinement	
*R*[*F* ^2^ > 2σ(*F* ^2^)], *wR*(*F* ^2^), *S*	0.057, 0.184, 1.06
No. of reflections	6073
No. of parameters	380
H-atom treatment	H atoms treated by a mixture of independent and constrained refinement
Δρ_max_, Δρ_min_ (e Å^−3^)	0.44, −0.41

Computer programs: *COLLECT* (Nonius, 1997 ▸), *HKL* *DENZO* and *SCALEPACK* (Otwinowski & Minor 1997 ▸), *SHELXS97* and *SHELXL97* (Sheldrick, 2008 ▸), *ORTEP-3 for Windows* and *WinGX* publication routines (Farrugia, 2012 ▸).

## full crystallographic data

### Crystal data

**Table:**

C_16_H_15_NO_4_	*Z* = 4
*M**_r_* = 285.29	*F*(000) = 600
Triclinic, *P*1	*D*_x_ = 1.420 Mg m^−^^3^
*a* = 8.3360 (2) Å	Mo *K*α radiation, λ = 0.71073 Å
*b* = 10.1780 (3) Å	Cell parameters from 11197 reflections
*c* = 16.3260 (4) Å	θ = 2.6–27.5°
α = 82.284 (2)°	µ = 0.10 mm^−^^1^
β = 76.557 (2)°	*T* = 293 K
γ = 87.081 (2)°	Prism, colourless
*V* = 1334.73 (6) Å^3^	0.36 × 0.23 × 0.15 mm

### Data collection

**Table:**

Nonius KappaCCD diffractometer	4671 reflections with *I* > 2σ(*I*)
Radiation source: Enraf Nonius FR590	*R*_int_ = 0.022
Horizonally mounted graphite crystal monochromator	θ_max_ = 27.5°, θ_min_ = 3.1°
Detector resolution: 9 pixels mm^-1^	*h* = −10→10
CCD rotation images,thick slices scans	*k* = −13→12
19726 measured reflections	*l* = −18→21
6073 independent reflections	

### Refinement

**Table:**

Refinement on *F*^2^	0 restraints
Least-squares matrix: full	Hydrogen site location: mixed
*R*[*F*^2^ > 2σ(*F*^2^)] = 0.057	H atoms treated by a mixture of independent and constrained refinement
*wR*(*F*^2^) = 0.184	*w* = 1/[σ^2^(*F*_o_^2^) + (0.1054*P*)^2^ + 0.3107*P*] where *P* = (*F*_o_^2^ + 2*F*_c_^2^)/3
*S* = 1.06	(Δ/σ)_max_ < 0.001
6073 reflections	Δρ_max_ = 0.44 e Å^−^^3^
380 parameters	Δρ_min_ = −0.40 e Å^−^^3^

### Special details

**Table:**

Geometry. All esds (except the esd in the dihedral angle between two l.s. planes) are estimated using the full covariance matrix. The cell esds are taken into account individually in the estimation of esds in distances, angles and torsion angles; correlations between esds in cell parameters are only used when they are defined by crystal symmetry. An approximate (isotropic) treatment of cell esds is used for estimating esds involving l.s. planes.
Refinement. All H atoms attached to C atoms were fixed geometrically and treated as riding with C—H = 0.93 Å (rings) or C—H = 0.96 Å (methyl snd hydroxy) with *U*_iso_(H) = 1.2*U*_eq_(C).

### Fractional atomic coordinates and isotropic or equivalent isotropic displacement parameters (Å^2^)

**Table:**

	*x*	*y*	*z*	*U*_iso_*/*U*_eq_	
O1	0.34832 (15)	0.29142 (11)	0.15710 (8)	0.0453 (3)	
O2	0.64583 (16)	0.21124 (12)	0.09702 (9)	0.0515 (3)	
O3	0.84531 (15)	0.65229 (13)	0.03086 (9)	0.0523 (3)	
O4	0.64390 (16)	0.83835 (12)	0.05781 (9)	0.0545 (3)	
O5	0.27751 (16)	0.74356 (12)	0.61638 (8)	0.0490 (3)	
O6	0.19210 (18)	0.86749 (12)	0.48348 (9)	0.0535 (3)	
O7	0.14839 (19)	0.46740 (13)	0.37138 (8)	0.0563 (4)	
O8	0.2317 (2)	0.25560 (13)	0.44761 (9)	0.0576 (4)	
N1	0.26613 (16)	0.57543 (13)	0.14686 (8)	0.0371 (3)	
N2	0.34120 (17)	0.45683 (13)	0.62919 (9)	0.0396 (3)	
C1	0.2369 (2)	0.71041 (16)	0.15183 (10)	0.0390 (4)	
C2	0.3625 (2)	0.80256 (16)	0.11976 (10)	0.0398 (4)	
C3	0.5293 (2)	0.75708 (16)	0.08543 (10)	0.0384 (3)	
C4	0.55708 (18)	0.61589 (15)	0.08865 (9)	0.0343 (3)	
C5	0.42538 (18)	0.52538 (15)	0.12017 (9)	0.0329 (3)	
C6	0.46427 (19)	0.38806 (15)	0.12420 (10)	0.0356 (3)	
C7	0.6265 (2)	0.34534 (16)	0.09288 (10)	0.0385 (3)	
C8	0.7552 (2)	0.43262 (17)	0.06184 (11)	0.0413 (4)	
H8	0.8624	0.4015	0.0425	0.050*	
C9	0.72055 (19)	0.56693 (16)	0.06034 (10)	0.0383 (3)	
C10	0.3281 (2)	0.93932 (18)	0.12005 (13)	0.0508 (4)	
H10	0.4120	0.9996	0.0974	0.061*	
C11	0.1715 (3)	0.9843 (2)	0.15353 (15)	0.0614 (5)	
H11	0.1481	1.0747	0.1525	0.074*	
C12	0.0491 (3)	0.8930 (2)	0.18875 (16)	0.0641 (6)	
H12	−0.0561	0.9233	0.2132	0.077*	
C13	0.0781 (2)	0.7589 (2)	0.18874 (13)	0.0538 (5)	
H13	−0.0066	0.7000	0.2130	0.065*	
C14	0.1194 (2)	0.49385 (19)	0.15926 (13)	0.0494 (4)	
H14A	0.0218	0.5462	0.1777	0.074*	
H14B	0.1163	0.4627	0.1067	0.074*	
H14C	0.1245	0.4194	0.2015	0.074*	
C15	0.3563 (3)	0.2314 (2)	0.24026 (12)	0.0567 (5)	
H15A	0.2732	0.1653	0.2599	0.085*	
H15B	0.4632	0.1907	0.2387	0.085*	
H15C	0.3378	0.2979	0.2781	0.085*	
C16	0.8075 (3)	0.1570 (2)	0.06854 (15)	0.0583 (5)	
H16A	0.8029	0.0620	0.0751	0.087*	
H16B	0.8493	0.1896	0.0098	0.087*	
H16C	0.8791	0.1829	0.1016	0.087*	
C17	0.28342 (19)	0.52898 (15)	0.56263 (10)	0.0360 (3)	
C18	0.2465 (2)	0.45975 (15)	0.49895 (10)	0.0375 (3)	
C19	0.2651 (2)	0.31822 (16)	0.50310 (11)	0.0418 (4)	
C20	0.3209 (2)	0.24810 (16)	0.57486 (11)	0.0403 (4)	
C21	0.3513 (2)	0.31925 (16)	0.63754 (10)	0.0389 (3)	
C22	0.2578 (2)	0.66750 (16)	0.55527 (10)	0.0394 (4)	
C23	0.2074 (2)	0.73401 (16)	0.48463 (11)	0.0425 (4)	
C24	0.1726 (2)	0.66747 (18)	0.42197 (11)	0.0461 (4)	
H24	0.1396	0.7139	0.3754	0.055*	
C25	0.1881 (2)	0.53179 (17)	0.43046 (11)	0.0422 (4)	
C26	0.3953 (3)	0.24636 (19)	0.70890 (12)	0.0522 (4)	
H26	0.4102	0.2903	0.7529	0.063*	
C27	0.4164 (3)	0.1110 (2)	0.71424 (14)	0.0600 (5)	
H27	0.4455	0.0647	0.7619	0.072*	
C28	0.3953 (3)	0.04215 (19)	0.64993 (15)	0.0587 (5)	
H28	0.4147	−0.0490	0.6531	0.070*	
C29	0.3454 (2)	0.11009 (18)	0.58159 (13)	0.0500 (4)	
H29	0.3276	0.0641	0.5391	0.060*	
C30	0.4188 (3)	0.5227 (2)	0.68466 (13)	0.0562 (5)	
H30A	0.4042	0.6170	0.6733	0.084*	
H30B	0.3684	0.4935	0.7430	0.084*	
H30C	0.5345	0.5005	0.6738	0.084*	
C31	0.1259 (3)	0.7728 (2)	0.67321 (14)	0.0658 (6)	
H31A	0.1471	0.8255	0.7139	0.099*	
H31B	0.0530	0.8210	0.6417	0.099*	
H31C	0.0755	0.6915	0.7022	0.099*	
C32	0.1415 (3)	0.94238 (19)	0.41295 (15)	0.0621 (5)	
H32A	0.1355	1.0349	0.4197	0.093*	
H32B	0.2201	0.9287	0.3614	0.093*	
H32C	0.0351	0.9139	0.4103	0.093*	
H1	0.7961	0.7434	0.0350	0.094 (6)*	
H2	0.1696	0.3765	0.3915	0.094 (6)*	

### Atomic displacement parameters (Å^2^)

**Table:**

	*U*^11^	*U*^22^	*U*^33^	*U*^12^	*U*^13^	*U*^23^
O1	0.0453 (7)	0.0403 (6)	0.0503 (7)	−0.0098 (5)	−0.0123 (5)	−0.0002 (5)
O2	0.0498 (7)	0.0375 (6)	0.0649 (8)	0.0037 (5)	−0.0077 (6)	−0.0100 (6)
O3	0.0331 (6)	0.0481 (7)	0.0706 (9)	−0.0067 (5)	−0.0031 (6)	−0.0028 (6)
O4	0.0450 (7)	0.0406 (6)	0.0731 (9)	−0.0082 (5)	−0.0079 (6)	0.0031 (6)
O5	0.0582 (8)	0.0420 (6)	0.0514 (7)	−0.0085 (5)	−0.0129 (6)	−0.0182 (5)
O6	0.0693 (9)	0.0340 (6)	0.0591 (8)	−0.0027 (6)	−0.0186 (6)	−0.0044 (5)
O7	0.0781 (9)	0.0519 (7)	0.0504 (7)	0.0021 (6)	−0.0344 (7)	−0.0144 (6)
O8	0.0878 (10)	0.0419 (7)	0.0548 (8)	−0.0002 (6)	−0.0334 (7)	−0.0177 (6)
N1	0.0306 (6)	0.0409 (7)	0.0388 (7)	−0.0026 (5)	−0.0078 (5)	−0.0009 (5)
N2	0.0459 (8)	0.0390 (7)	0.0380 (7)	−0.0055 (6)	−0.0149 (6)	−0.0085 (5)
C1	0.0374 (8)	0.0434 (8)	0.0370 (8)	0.0038 (6)	−0.0117 (6)	−0.0043 (6)
C2	0.0404 (8)	0.0395 (8)	0.0412 (8)	0.0028 (6)	−0.0153 (7)	−0.0024 (6)
C3	0.0383 (8)	0.0394 (8)	0.0376 (8)	−0.0021 (6)	−0.0119 (6)	0.0010 (6)
C4	0.0337 (7)	0.0385 (8)	0.0312 (7)	−0.0013 (6)	−0.0109 (6)	−0.0002 (6)
C5	0.0322 (7)	0.0392 (8)	0.0283 (7)	−0.0010 (6)	−0.0101 (6)	−0.0019 (6)
C6	0.0363 (8)	0.0372 (8)	0.0341 (7)	−0.0046 (6)	−0.0101 (6)	−0.0018 (6)
C7	0.0424 (8)	0.0379 (8)	0.0372 (8)	0.0029 (6)	−0.0126 (7)	−0.0064 (6)
C8	0.0334 (8)	0.0471 (9)	0.0430 (9)	0.0029 (7)	−0.0084 (6)	−0.0063 (7)
C9	0.0322 (7)	0.0443 (8)	0.0378 (8)	−0.0033 (6)	−0.0082 (6)	−0.0026 (6)
C10	0.0532 (10)	0.0410 (9)	0.0624 (11)	0.0042 (7)	−0.0237 (9)	−0.0045 (8)
C11	0.0575 (12)	0.0487 (10)	0.0840 (15)	0.0164 (9)	−0.0267 (11)	−0.0177 (10)
C12	0.0476 (11)	0.0653 (13)	0.0804 (14)	0.0181 (9)	−0.0140 (10)	−0.0214 (11)
C13	0.0393 (9)	0.0592 (11)	0.0614 (11)	0.0042 (8)	−0.0078 (8)	−0.0106 (9)
C14	0.0335 (8)	0.0531 (10)	0.0605 (11)	−0.0079 (7)	−0.0105 (7)	−0.0011 (8)
C15	0.0694 (13)	0.0494 (10)	0.0464 (10)	−0.0116 (9)	−0.0058 (9)	0.0018 (8)
C16	0.0537 (11)	0.0490 (10)	0.0731 (13)	0.0145 (8)	−0.0156 (10)	−0.0147 (9)
C17	0.0356 (8)	0.0378 (8)	0.0353 (8)	−0.0056 (6)	−0.0069 (6)	−0.0072 (6)
C18	0.0398 (8)	0.0375 (8)	0.0373 (8)	−0.0032 (6)	−0.0099 (6)	−0.0096 (6)
C19	0.0465 (9)	0.0404 (8)	0.0418 (8)	−0.0040 (7)	−0.0123 (7)	−0.0116 (7)
C20	0.0391 (8)	0.0390 (8)	0.0447 (9)	−0.0030 (6)	−0.0111 (7)	−0.0081 (7)
C21	0.0364 (8)	0.0403 (8)	0.0413 (8)	−0.0038 (6)	−0.0098 (6)	−0.0070 (7)
C22	0.0423 (8)	0.0363 (8)	0.0413 (8)	−0.0071 (6)	−0.0087 (7)	−0.0104 (6)
C23	0.0429 (9)	0.0359 (8)	0.0482 (9)	−0.0041 (6)	−0.0080 (7)	−0.0067 (7)
C24	0.0517 (10)	0.0442 (9)	0.0443 (9)	−0.0001 (7)	−0.0159 (8)	−0.0037 (7)
C25	0.0461 (9)	0.0445 (9)	0.0397 (8)	−0.0015 (7)	−0.0140 (7)	−0.0113 (7)
C26	0.0618 (11)	0.0513 (10)	0.0489 (10)	−0.0043 (8)	−0.0242 (9)	−0.0039 (8)
C27	0.0666 (13)	0.0526 (11)	0.0653 (12)	−0.0021 (9)	−0.0310 (10)	0.0046 (9)
C28	0.0628 (12)	0.0400 (9)	0.0783 (14)	0.0001 (8)	−0.0296 (11)	−0.0018 (9)
C29	0.0521 (10)	0.0411 (9)	0.0627 (11)	−0.0006 (7)	−0.0213 (9)	−0.0126 (8)
C30	0.0723 (13)	0.0528 (11)	0.0552 (11)	−0.0033 (9)	−0.0324 (10)	−0.0163 (9)
C31	0.0763 (14)	0.0600 (12)	0.0560 (12)	−0.0111 (10)	0.0046 (10)	−0.0200 (10)
C32	0.0764 (14)	0.0402 (9)	0.0699 (13)	0.0006 (9)	−0.0228 (11)	0.0021 (9)

### Geometric parameters (Å, º)

**Table:**

O1—C6	1.3798 (19)	C13—H13	0.9300
O1—C15	1.427 (2)	C14—H14A	0.9600
O2—C7	1.3606 (19)	C14—H14B	0.9600
O2—C16	1.428 (2)	C14—H14C	0.9600
O3—C9	1.347 (2)	C15—H15A	0.9600
O3—H1	1.00	C15—H15B	0.9600
O4—C3	1.257 (2)	C15—H15C	0.9600
O5—C22	1.3849 (19)	C16—H16A	0.9600
O5—C31	1.427 (2)	C16—H16B	0.9600
O6—C23	1.356 (2)	C16—H16C	0.9600
O6—C32	1.429 (2)	C17—C22	1.408 (2)
O7—C25	1.345 (2)	C17—C18	1.428 (2)
O7—H2	0.96	C18—C25	1.421 (2)
O8—C19	1.263 (2)	C18—C19	1.435 (2)
N1—C5	1.3905 (19)	C19—C20	1.447 (2)
N1—C1	1.393 (2)	C20—C29	1.402 (2)
N1—C14	1.473 (2)	C20—C21	1.405 (2)
N2—C21	1.389 (2)	C21—C26	1.408 (2)
N2—C17	1.395 (2)	C22—C23	1.397 (2)
N2—C30	1.472 (2)	C23—C24	1.390 (2)
C1—C2	1.401 (2)	C24—C25	1.372 (2)
C1—C13	1.414 (2)	C24—H24	0.9300
C2—C10	1.407 (2)	C26—C27	1.374 (3)
C2—C3	1.450 (2)	C26—H26	0.9300
C3—C4	1.440 (2)	C27—C28	1.386 (3)
C4—C9	1.421 (2)	C27—H27	0.9300
C4—C5	1.425 (2)	C28—C29	1.369 (3)
C5—C6	1.415 (2)	C28—H28	0.9300
C6—C7	1.399 (2)	C29—H29	0.9300
C7—C8	1.386 (2)	C30—H30A	0.9600
C8—C9	1.381 (2)	C30—H30B	0.9600
C8—H8	0.9300	C30—H30C	0.9600
C10—C11	1.374 (3)	C31—H31A	0.9600
C10—H10	0.9300	C31—H31B	0.9600
C11—C12	1.384 (3)	C31—H31C	0.9600
C11—H11	0.9300	C32—H32A	0.9600
C12—C13	1.374 (3)	C32—H32B	0.9600
C12—H12	0.9300	C32—H32C	0.9600
			
C6—O1—C15	113.10 (13)	O2—C16—H16A	109.5
C7—O2—C16	118.42 (15)	O2—C16—H16B	109.5
C9—O3—H1	106.7	H16A—C16—H16B	109.5
C22—O5—C31	113.22 (15)	O2—C16—H16C	109.5
C23—O6—C32	117.70 (15)	H16A—C16—H16C	109.5
C25—O7—H2	101.9	H16B—C16—H16C	109.5
C5—N1—C1	120.95 (13)	N2—C17—C22	123.21 (14)
C5—N1—C14	122.23 (14)	N2—C17—C18	118.85 (14)
C1—N1—C14	116.26 (13)	C22—C17—C18	117.94 (15)
C21—N2—C17	121.03 (13)	C25—C18—C17	119.60 (15)
C21—N2—C30	116.86 (14)	C25—C18—C19	119.06 (14)
C17—N2—C30	121.38 (14)	C17—C18—C19	121.34 (15)
N1—C1—C2	121.35 (14)	O8—C19—C18	122.04 (16)
N1—C1—C13	120.78 (16)	O8—C19—C20	120.53 (15)
C2—C1—C13	117.87 (16)	C18—C19—C20	117.42 (14)
C1—C2—C10	120.56 (16)	C29—C20—C21	120.19 (16)
C1—C2—C3	119.91 (15)	C29—C20—C19	120.23 (15)
C10—C2—C3	119.53 (16)	C21—C20—C19	119.58 (14)
O4—C3—C4	122.44 (15)	N2—C21—C20	121.49 (15)
O4—C3—C2	120.66 (15)	N2—C21—C26	120.81 (15)
C4—C3—C2	116.82 (14)	C20—C21—C26	117.69 (16)
C9—C4—C5	119.82 (14)	O5—C22—C23	116.96 (14)
C9—C4—C3	118.60 (14)	O5—C22—C17	123.00 (15)
C5—C4—C3	121.57 (14)	C23—C22—C17	120.03 (14)
N1—C5—C6	123.14 (14)	O6—C23—C24	123.22 (16)
N1—C5—C4	118.88 (14)	O6—C23—C22	114.49 (15)
C6—C5—C4	117.96 (14)	C24—C23—C22	122.28 (15)
O1—C6—C7	117.14 (14)	C25—C24—C23	118.45 (16)
O1—C6—C5	123.08 (14)	C25—C24—H24	120.8
C7—C6—C5	119.78 (14)	C23—C24—H24	120.8
O2—C7—C8	123.57 (15)	O7—C25—C24	118.28 (15)
O2—C7—C6	113.88 (14)	O7—C25—C18	120.19 (15)
C8—C7—C6	122.54 (15)	C24—C25—C18	121.53 (15)
C9—C8—C7	118.40 (15)	C27—C26—C21	120.69 (17)
C9—C8—H8	120.8	C27—C26—H26	119.7
C7—C8—H8	120.8	C21—C26—H26	119.7
O3—C9—C8	118.70 (14)	C26—C27—C28	121.28 (18)
O3—C9—C4	119.92 (15)	C26—C27—H27	119.4
C8—C9—C4	121.38 (15)	C28—C27—H27	119.4
C11—C10—C2	120.43 (19)	C29—C28—C27	119.12 (18)
C11—C10—H10	119.8	C29—C28—H28	120.4
C2—C10—H10	119.8	C27—C28—H28	120.4
C10—C11—C12	119.01 (18)	C28—C29—C20	120.86 (17)
C10—C11—H11	120.5	C28—C29—H29	119.6
C12—C11—H11	120.5	C20—C29—H29	119.6
C13—C12—C11	121.98 (18)	N2—C30—H30A	109.5
C13—C12—H12	119.0	N2—C30—H30B	109.5
C11—C12—H12	119.0	H30A—C30—H30B	109.5
C12—C13—C1	120.04 (19)	N2—C30—H30C	109.5
C12—C13—H13	120.0	H30A—C30—H30C	109.5
C1—C13—H13	120.0	H30B—C30—H30C	109.5
N1—C14—H14A	109.5	O5—C31—H31A	109.5
N1—C14—H14B	109.5	O5—C31—H31B	109.5
H14A—C14—H14B	109.5	H31A—C31—H31B	109.5
N1—C14—H14C	109.5	O5—C31—H31C	109.5
H14A—C14—H14C	109.5	H31A—C31—H31C	109.5
H14B—C14—H14C	109.5	H31B—C31—H31C	109.5
O1—C15—H15A	109.5	O6—C32—H32A	109.5
O1—C15—H15B	109.5	O6—C32—H32B	109.5
H15A—C15—H15B	109.5	H32A—C32—H32B	109.5
O1—C15—H15C	109.5	O6—C32—H32C	109.5
H15A—C15—H15C	109.5	H32A—C32—H32C	109.5
H15B—C15—H15C	109.5	H32B—C32—H32C	109.5

### Hydrogen-bond geometry (Å, º)

**Table:**

*D*—H···*A*	*D*—H	H···*A*	*D*···*A*	*D*—H···*A*
C8—H8···O3^i^	0.93	2.52	3.441 (2)	170
C32—H32*A*···O8^ii^	0.96	2.55	3.460 (3)	157
C30—H30*C*···O7^iii^	0.96	2.59	3.513 (3)	159

Symmetry codes: (i) −*x*+2, −*y*+1, −*z*; (ii) *x*, *y*+1, *z*; (iii) −*x*+1, −*y*+1, −*z*+1.
